# Intranasally administered protein coated chitosan nanoparticles encapsulating influenza H9N2 HA2 and M2e mRNA molecules elicit protective immunity against avian influenza viruses in chickens

**DOI:** 10.1186/s13567-020-00762-4

**Published:** 2020-03-06

**Authors:** Irshad Ahmed Hajam, Amal Senevirathne, Chamit Hewawaduge, Jehyoung Kim, John Hwa Lee

**Affiliations:** College of Veterinary Medicine, Jeonbuk National University, Iksan, 54596 Republic of Korea

## Abstract

Chitosan nanoparticles (CNPs) represent an efficient vaccination tool to deliver immunogenic antigens to the antigen-presenting cells (APCs), which subsequently stimulate protective immune responses against infectious diseases. Herein, we prepared CNPs encapsulating mRNA molecules followed by surface coating with conserved H9N2 HA2 and M2e influenza proteins. We demonstrated that CNPs efficiently delivered mRNA molecules into APCs and had effectively penetrated the mucosal barrier to reach to the immune initiation sites. To investigate the potential of CNPs delivering influenza antigens to stimulate protective immunity, we intranasally vaccinated chickens with empty CNPs, CNPs delivering HA2 and M2e in both mRNA and protein formats (CNPs + RNA + Pr) or CNPs delivering antigens in protein format only (CNPs + Pr). Our results demonstrated that chickens vaccinated with CNPs + RNA + Pr elicited significantly (*p* < 0.05) higher systemic IgG, mucosal IgA antibody responses and cellular immune responses compared to the CNPs + Pr vaccinated group. Consequently, upon challenge with either H7N9 or H9N2 avian influenza viruses (AIVs), efficient protection, in the context of viral load and lung pathology, was observed in chickens vaccinated with CNPs + RNA + Pr than CNPs + Pr vaccinated group. In conclusion, we show that HA2 and M2e antigens elicited a broad spectrum of protection against AIVs and incorporation of mRNAs in vaccine formulation is an effective strategy to induce superior immune responses.

## Introduction

Avian influenza viruses (AIVs), broadly designated as highly pathogenic avian influenza (HPAI) or low pathogenic avian influenza (LPAI), annually cause significant economic losses in the poultry industry worldwide [1, 2]. Outbreaks of LPAI viruses belonging to H7 and H9 subtypes in chickens have been reported in the past [3–6] and infections caused by H7N9 and H9N2 LPAI viruses have not only infected poultry birds but also demonstrated their potential to infect humans [7, 8]. The H9N2 virus is the most prevalent LPAI subtype circulating endemically in poultry across Asia, the Middle East and Northern Africa [9, 10]. Despite being primarily a poultry pathogen, the H9N2 virus has been reported to diversify the host range and conferring a zoonotic transmission potential to H7N9 and H10N8 viruses, which are reported to cause deadly human infections [6, 11, 12]. Thus, the H9N2 influenza subtype can lead to the emergence of novel reassortants with the ability to cause potential pandemics. Therefore, the development of novel strategies and/or efficient vaccines to confine this infection has immense public health importance. In addition to strict biosecurity measures, the H9N2 virus has been controlled mainly through the use of oil adjuvanted inactivated whole H9N2 virus vaccine in South Korea [13]. Although vaccination has drastically reduced the incidence of H9N2 infection in chicken farms, this oil adjuvanted H9N2 vaccine requires a large supply of specific-pathogen-free (SPF) embryonated eggs, which is a time-consuming process and generally takes 6 to 8 months for the production of a new vaccine. Hence, the preparation of a new vaccine during epidemic/pandemic situations is difficult. Furthermore, because of vaccination and subsequent selective immune pressure, the H9N2 virus has been continuously evolving through antigenic drift [14], thus making the currently available H9N2 vaccines inefficient. Therefore, subunit-based vaccines that are easy to manipulate, produce, and scale-up and targeting conserved epitopes that are known to stimulate a broad spectrum of protection would be the ideal choice for controlling outbreaks caused by emergent pandemic strains.

Hemagglutinin (HA) is the most abundant integral viral envelope protein and is the major target for generating protective immunity. HA is synthesized as a precursor protein (HA0) that is cleaved by cellular proteases into HA1 and HA2 subunits, a process that is essential to convert HA0 into a fusion active form [15]. HA2 region is considerably more conserved than HA1, and, in recent years, several attempts have been made to develop universal influenza vaccines based on HA2 and the interacting portions of HA1 [16–18]. These studies have demonstrated that HA2-directed neutralizing antibodies can provide a broad range of protection against lethal doses of homo- and heterosubtypic influenza A virus challenges. Similar to HA, matrix protein 2 (M2) of influenza A virus is an integral transmembrane protein and its ectodomain (M2e) is considered a promising candidate antigen to elicit heterologous protection [19]. While natural infections and currently available conventional influenza A virus vaccines generally induce very low M2e-specific humoral responses [20], presenting M2e on a suitable carrier or linking several copies of M2e in tandem greatly enhances its immunogenicity and cross-protective potential [21]. Considering that both M2e and HA2 are highly conserved influenza proteins and their potential to stimulate a broad spectrum of protection, we, in this study, have delivered M2e and HA2 antigens via chitosan nanoparticles (CNPs) to elicit protective immune responses against LPAI viruses. To generate a mucosal vaccine targeting the nasal route of immunization, CNPs could be a plausible choice to enhance antigen retention upon the mucosal surface. The positively charged CNPs will promote the adhesion onto the negatively charged nasal mucosal surfaces and thus promote the retention and presentation of bound antigens into the antigen-presenting cells such as macrophages and dendritic cells.

Recently, mRNA-based vaccines against infectious diseases have been developed, and such studies have demonstrated the potential of these vaccines to elicit strong humoral and cell-mediated immunity, and complete protection against the lethal challenges [22, 23]. The mRNA-based vaccines are highly advantageous over viral-based replicons or DNA vaccines as they are easy to produce and can mitigate the risk of insertional mutagenesis. In accordance with this notion, we hypothesize that coadministering antigens in both mRNA and protein formats will increase the efficacy of subunit-based vaccines. In this study, we attempted to mimic the structure of an influenza virus by encapsulating mRNA in the core and HA2 and M2e proteins on the periphery by using chitosan nanoparticles. We coadministered HA2 and M2e antigens in mRNA and protein formats using chitosan, a biopolymer of glucosamine residues, nanoparticles as a delivery vehicle. CNPs are widely used as a delivery system for drugs and vaccine antigens [24, 25]. Because of surface charge and hydrophobicity, CNPs effectively target vaccine antigens to the antigen-presenting cells (APCs), such as macrophages and dendritic cells (DCs) [26], which subsequently activate efficient antigen-specific T cell responses, necessary for the confinement of pathogenic infections [27]. Moon et al. demonstrated that intranasal administration of CNPs delivering HA1 protein elicited superior humoral and cell-mediated immunity than immunization with the whole-inactivated influenza vaccine [28]. In the present study, based on the study of Bommakanti et al. [16], we designed H9N2 HA2 immunogen along with the interacting residues of the HA1 subunit so that the recombinant HA2 subunit would get expressed in a soluble form in a prokaryotic expression system, and the resulting construct was termed HA2-HA1 immunogen. We show that intranasal immunization with CNPs delivering HA2-HA1 and M2e antigens in both protein and mRNA formats elicited superior protective immune responses against H7N9 and H9N2 LPAI viruses compared to the CNPs delivering HA2-HA1 and M2e antigens in a protein format only.

## Materials and methods

### Virus and cell line

The tissue culture infective dose (TCID_50_) of H7N9 and H9N2 AIVs, cultivated in the allantoic cavities of SPF embryonated eggs, was calculated in Madin Darby Canine Kidney (MDCK) cells, as described previously [29]. The THP-1 (human monocytic leukemia) cell line used for the in vitro mRNA expression studies of HA2 and M2e antigens was cultured in RPMI media with 10% fetal bovine serum (FBS) and antibiotics, penicillin and streptomycin.

### Expression and purification of H9N2 HA2-HA1 and M2e proteins

The design of the conserved H9N2 HA2 immunogen was constructed based on the interacting residues of HA1 with the HA2 subunit, by adopting a previously described method [16]. The designated protein contained residues of HA2 (1–172), a 7-amino acid linker (GSAGSAG), HA1 residues (19–80), a 6-amino acid linker (GSAGSA) followed by residues (287–338) of HA1. The gene sequence was codon-optimized for the *Salmonella* system and then synthesized and built into the prokaryotic expression vector pET28a (+) (Novagen, San Diego, USA). The recombinant pET28a-HA2-HA1 plasmid was subsequently transformed into *E. coli* BL21 (DE3) pLysS host strain (Novagen, San Diego, USA) for expression and purification of HA2-HA1 recombinant protein, as described previously [30]. The four tandem repeats of conserved M2e gene sequence (MSLLTEVETPTRNGWECKCSDSSD) were cloned in-frame into pET32a (+) prokaryotic expression vector (Novagen, San Diego, USA) and expressed in *E. coli* BL21 (DE3) pLysS host strain (Novagen, San Diego, USA), as discussed and reported elsewhere [31].

### HA2 and M2e mRNA synthesis

H9N2 HA2 or M2e mRNA was synthesized in vitro using T7 polymerase-mediated DNA-dependent RNA transcription using the T7 RNA polymerase kit (#M0251S), followed by capping (#M2080S) and polyA tailing (#M0276S) as previously described [22]. The mRNA contained cap 0 (7-methylguanylate), 5ʹUTR (GGGAAAUAAGAGAGAAAAGAAGAGUAAGAAGAAAUAUAAGAGCCACC), signal sequence from human IgE (MDWTWILFLVAAATRVHS), HA2/M2e gene sequence, 3`UTR (UGAUAAUAGGCUGGAGCCUCGGUGGCCAU) and polyA tail. The synthesized mRNA was stored at −80 °C until further use.

### Preparation and characterization of protein-coated CNPs encapsulating HA2 and M2e mRNAs

The protein-coated CNPs encapsulating mRNAs were prepared by an anionic gelation method [32]. The 1% (w/v) solution of low molecular weight chitosan (Sigma-Aldrich) was prepared by slowly dissolving chitosan particles in an aqueous solution of 4% acetic acid under magnetic stirring until the solution became transparent. The solution was then subjected to sonication using a 20 kHz Sonicator (QSONICA-Part No. Q500, USA) and the sonication process was run for 10 min on ice. The pH was adjusted to 4.5 and the so-obtained CNP solution was filtered through a 0.2 μm syringe. To prepare CNPs encapsulating HA2 and M2e mRNAs, 1 mL of 1% CNP solution was added to 1 mL of deionized water and incubated with 60 µg of each HA2 and M2e mRNAs at room temperature (RT) for 10 min. Subsequently, 0.5 mL of 1% (w/v) sodium tripolyphosphate (TPP) (Sigma-Aldrich) in 0.5 mL deionized water was added into the solution and subjected to slow stirring at RT for 20 min. For surface coating, 1 mg of each HA2-HA1 and M2e proteins in PBS was added to the solution and centrifuged at 10 500 × *g* for 10 min to collect chitosan-mRNA-protein particles, which were subsequently washed twice with PBS and then the pellet was finally dissolved in 1.5 mL of PBS. The size and morphological characteristics of the prepared CNPs were visualized under Field Emission Scanning Electron Microscopy (FESEM; Zeiss Supra 40VP) and Transmission Electron Microscopy (TEM; Hitachi H-7650) at 100K magnification, as previously described [33]. The particle size distribution was estimated using 100 particles measured in a random manner using image analysis software. The CNP surface bound proteins were estimated using Bradford assay for individual protein interacting with CNPs [34] and an ELISA-based assay for the determination of the ratio of each protein (to be described elsewhere). For in vitro and in vivo particle uptake studies, rhodamine B isothiocyanate (RITC) labelled empty or protein coated CNPs were prepared by incubation with 1.25 mg RITC dye (Sigma-Aldrich) for 5 min, as described previously [35]. The formulated fluorescently tagged CNPs were separated by centrifugation at 10 500 × *g* for 5 min and stored at 4 °C until further use.

For analysis of HA2 and M2e mRNAs expressions in vitro, 1 × 10^5^ THP-1 cells were seeded in a 96-well tissue culture plate and treated with 25 µL of either empty CNP solution (approximately 250 µg of CNPs), CNPs prepared with HA2 mRNA (1 µg mRNA/well), or CNPs prepared with M2e mRNA (1 µg mRNA/well) for 18 h. Post-incubation, the cells were washed thrice with sterile PBS and subsequently fixed and permeabilized using fixation and permeabilization kits (#eBioscience). To confirm HA2 and M2e expressions, the cells were treated with either rabbit polyclonal anti-HA antibody (#MBS270809) or polyclonal anti-M2 (#MBS9405612) antibody, followed by labeling of cells with AlexaFluor™ 488 donkey anti-rabbit IgG (Invitrogen). The green fluorescence, indicative of expression, exhibited by cells was finally visually under confocal microscopy (Carl Zeiss, Germany). As a positive control, we delivered mRNA molecules (1 µg mRNA/well) into THP-1 cells using LyoVec™ (InvivoGen, San Diego, USA) according to the manufacturer’s instructions.

### In vitro and in vivo safety evaluation of CNPs

For in vitro safety evaluation, 2 mL of blood was drawn from the jugular vein of 4 weeks old female layer chickens collected in 3 mL syringes preloaded with 0.02 mL of 1% potassium EDTA. The blood was centrifuged at 1000 × *g* for 5 min, and the RBC pellet was washed thrice with sterile PBS before resuspension in 3 mL PBS. To assess the impact of CNPs on the RBC lysis, 100 µL of RBCs was treated with different concentrations of CNPs ranging from 32.25 to 500 µg for 1 h at RT. Triton x-100 and PBS treated RBCs were kept as positive and negative controls, respectively. After incubation, the treated RBCs were centrifuged at 10 000 × *g* for 5 min and the supernatant containing released hemoglobin was measured (OD_575nm_) using the Infinite_M200_ NanoQuant (Tecan). The hemolysis was expressed in percentage using the following formula: [(sample absorbance − negative control)/(positive control − negative control)] × 100%. In addition, morphological observation of RBC interaction with CNPs was observed under the microscope and the integrity of cells was evaluated. Herein, 10^5^ red blood cells/well were seeded and interacted with 250 µg and 32 µg of CNP per well. 0.1% Triton X-100 treated cells were considered as a positive control. After 1 h incubation, cells were subjected to microscopic examination (Leica, Germany). To investigate the in vivo effect of CNPs on lung cells, chickens (*n* = 3) were intranasally administered with 60 µL of CNP solution, and 5 days later, the lung tissues were aseptically isolated for histological analysis, as previously described [36].

### In vitro and in vivo particle uptake assays

To investigate the ability of macrophages to take up CNPs, chicken bone marrow-derived macrophages were prepared and cultured in the presence of GM-CSF cytokines, as previously described [37]. 1 × 10^5^ macrophage cells were seeded in a 96-well tissue culture plate and incubated for 4 h at 37 °C. Chitosan nanoparticles were prepared as previously described and labeled with Rhodamine isothiocyanate (RITC) dye via the ionic gelation procedure. After adding TPP into the suspension, RITC entrapped CNPs were generated. The nanoparticles were harvested by centrifugation at 7500 RPM for 10 min and washed twice with PBS. The resultant nanoparticle coagulate was then ground to a fine powder using liquid nitrogen. The powder was dried at 37 °C in a desiccator and these particles were resuspended in 1 mL PBS and 20 µL was incubated with cultured macrophage cells for 17 h. The cell-internalized nanoparticles emitting the Rhodamine signal were observed under the microscope (Leica, Germany).

To assess the degree of chitosan nanoparticles attachment into the nasopharyngeal mucosal surfaces, 4-week old female layer chickens (*n* = 3) were intranasally inoculated with RITC labeled chitosan nanoparticle formulation (200 µL/bird/nostril from 10 mg/mL stock in PBS) into the left nostril of each chicken drop-wise over a long period for slow uptake into the nasal cavity. One, three and four hours after inoculation, the chickens were sacrificed and tissue sections across the upper nasal cavity were retrieved by slicing across the beak area revealing the fine structures of the nasal cavity. Tissues were preserved in 10% formaldehyde (PBS) for 3 days and decalcified over a week. The processed tissues were then embedded in paraffin and processed for immunohistochemical assessment. Five micrometer thin sections were stained with methyl green and the presence of nanoparticles was observed under the fluorescent microscope using Rhodamine filter.

### Protein uptake efficacy of chitosan

To assess the adsorption efficacy of each HA2-HA1 and M2e protein, a calibration curve was developed using BSA standard, 50–1000 ng of proteins. The quantity of proteins was determined by the Bradford assay. A graph was plotted using the absorbance versus protein concentration using microassay platform. A linear curve was fitted in the linear region of the graph and used for bound protein quantification. The quantities of each HA2-HA1 and M2e proteins individually bound on CNPs were determined by the Bradford assay using 20 µL (200 µg) of CNP preparation. After color measurement at 595 nm wave length, corresponding protein concentration was determined using the calibration curve. To assess protein adsorption with CNPs when a combination of proteins was used at 1 mg/mL concentration, CNPs were prepared using a combination of HA2-HA1 and M2e proteins as described earlier and re-suspended in 1 mL of PBS. Twenty microliters of chitosan were coated on ELISA plates in NaHCO_3_ coating buffer and blocked with 5% bovine serum albumin (BSA). After overnight incubation at 4 °C, each well was washed thrice with 0.01% PBST and reacted with each antigen-specific antibody. After color development, by adding the O-Phenylenediamine dihydrochloride substrate, the signal intensity was measured at 490 nm wavelength. The ratio of absorbance and the corresponding bound protein concentration was enumerated using the calibration curve previously described (Additional file 1).

### Immunization and challenge studies

All animal experimentation work was approved by the Chonbuk National University Animal Ethics Committee (CBU 2014-1-0038), and the chicken experiments were carried out according to the guidelines of the Korean Council on Animal Care. One-day-old female layer chickens (Corporation of Join hatchery, Republic of Korea) were maintained under standard conditions and provided antibiotic-free food and water ad libitum. Four weeks later, the chickens were randomly divided into three groups (*n *= 17 in each group) and vaccinated intranasally with empty CNPs, HA2-HA1 (33 µg) plus M2e (33 µg) coated CNPs (CNP + Pr) or CNP + Pr + 4 µg mRNAs of each HA2 and M2e (CNP + RNA + Pr) in a volume of 100 µL. Three weeks later, the chickens were boosted intranasally with the same dose. One week post-booster vaccination, all the vaccinated and the control chickens were intranasally challenged with 10^4^ TCID_50_ of either H7N9 or H9N2 virus and the cloacal swabs were collected post-challenge to determine the viral load by the qRT-PCR assay, as described previously [36]. For histopathological analysis, six chickens in each group were sacrificed on day 8 post-challenge and lung tissues were aseptically collected for histopathological analysis, as previously described [36].

### Systemic IgG and mucosal IgA specific antibody responses

Blood (*n* = 6) was drawn from the jugular vein of vaccinated and control birds on 14^th^, and 28^th^-day post-first vaccination and serum were separated to assess the systemic IgG responses by an indirect ELISA. For IgA analysis, five chickens in each group were sacrificed on day 28^th^ post-first vaccination and lung washings were collected to determine the HA2 and M2e specific IgA responses [36]. Purified 250 ng/well of HA2 or M2e protein was used as a coating antigen to determine antigen-specific humoral responses in an indirect ELISA.

### Neutralization assay

Neutralizing activity of the immunized sera against the H7N9 or H9N2 virus was measured by the micro-neutralization assay as previously described [36]. One week later, post H7N9 or H9N2 challenge, blood (*n* = 5) was drawn from vaccinated and control birds on day 7 post-challenge and sera were subsequently isolated for the determination of serum neutralizing antibody titers. The serum neutralization titers are calculated as log_2_ of the reciprocal of the last serum dilution that neutralized either H7N9 or H9N2 virus activity by 50%.

### Cell-mediated immune responses

A week after booster inoculation, peripheral blood mononuclear cells (PBMCs) were harvested using the wing vein of chicken (*n* = 6). The PBMCs were harvested by Histopaque density gradient centrifugation and seeded in either at 10^5^ cells/well in 96-well plates for lymphocyte proliferation assay or in 24-well plates for flow cytometry analysis. Proliferation response of PBMC following antigen stimulation (each HA2-HA1 and M2e at 300 ng/well concentration) in vitro was assayed by using 3-(4,5-dimethylthiazol-2-yl)-2, 5-diphenyl tetrazolium bromide (MTT) after 3 days of incubation. MTT formazan product was determined using a microplate reader at an absorbance of 570 nm. Besides, changes in T cell population response upon immunization were analyzed using PBMC seeded at 1 × 10^5^ in 24-well plates. Cells were then stimulated with each antigen (300 ng/well) or RPMI media alone for 72 h. Cells were stained with FITC anti-chicken CD3+, AF-700 anti-chicken CD4+ and PE anti-chicken PE CD8+ (Miltenyi Biotec, Bergisch Gladbach, Germany) for 30 min on ice. The analysis was carried out using a Macsquant fluorescent assisted cell sorting system (FACS) (Mieltenyi, Germany) instrument. Data analysis was performed using Macsquant (Miltenyi, Germany).

### Statistical analysis

All the obtained data were analyzed using GraphPad Prism 6.00 program (San Diego, CA, USA). Statistical significance was determined by one-way ANOVA (with Tukey’s multiple comparisons tests). *p* values of < 0.05 were considered statistically significant.

## Results

### Purification of the recombinant proteins

The HA2-HA1 protein expressed in *E. coli* BL21 host strain was purified by Ni–NTA column chromatography and analyzed by SDS-PAGE and western blotting (Additional file 2). SDS-PAGE analysis revealed that the purity of HA2-HA1 recombinant protein was > 95% as a single band of approximately 39 kDa size was observed (Additional file 2A). Western blotting indicated that HA2-HA1 protein reacted specifically with the H9N2 polyclonal anti-HA antibody giving a characteristic band at the expected size, thus confirming the authenticity of the expressed protein (Additional file 2B). The expression of the conserved H9N2 M2e tandem repeat sequence is described and reported elsewhere [31].

### Characterization of HA2 and M2e surface coated CNPs

**Figure 1:**
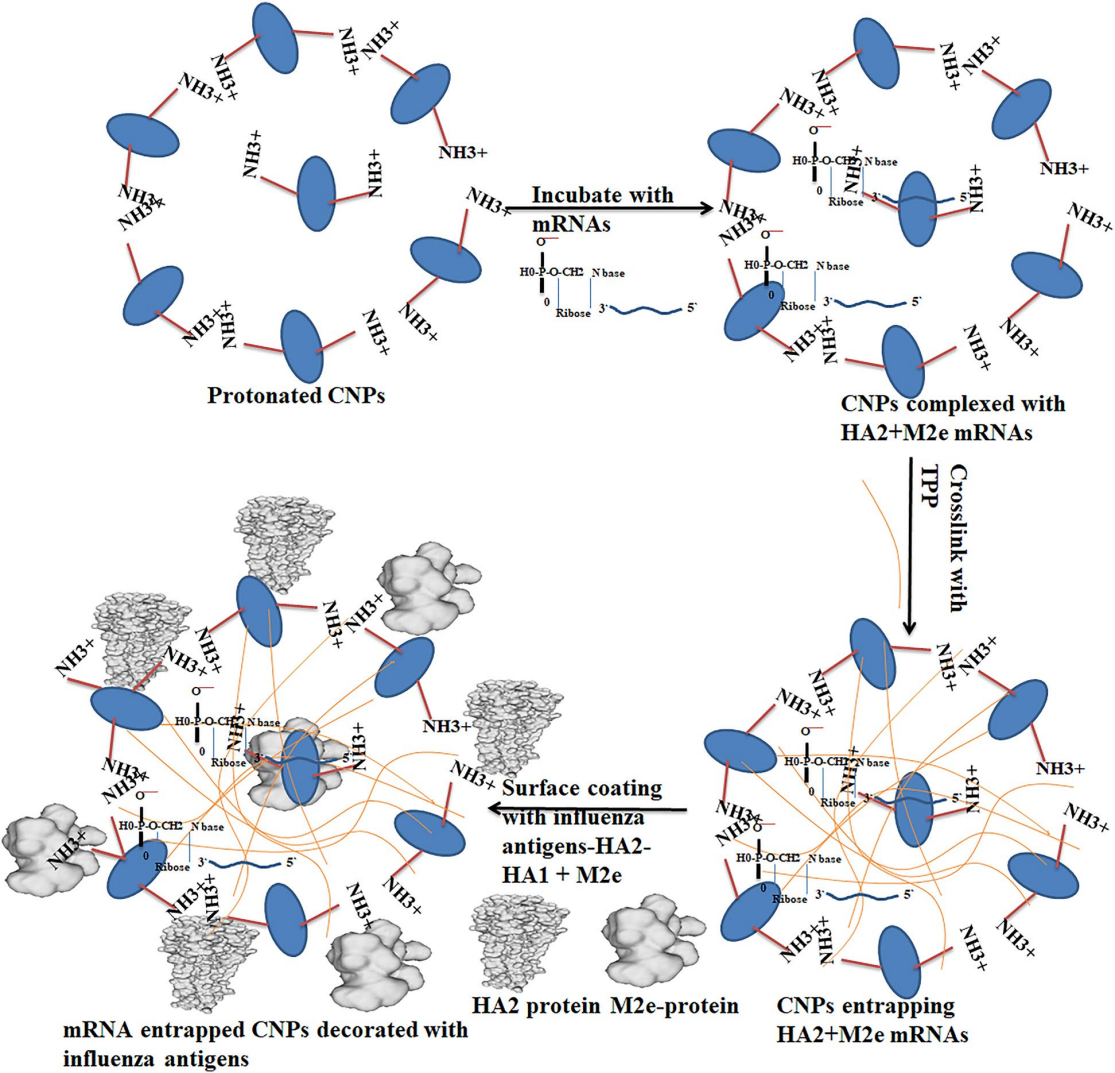
**Schematic illustration of the formulation of chitosan-based vaccine delivering influenza antigens in both protein and mRNA formats.** At pH 4.5, chitosan amine groups are positively charged and can form stable interactions with the negatively charged phosphate groups of mRNA molecules. The addition of TPP will help in entrapment of mRNA molecules inside CNPs and as well as the binding of the negatively charged proteins to the surface of the CNPs.

**Figure 2:**
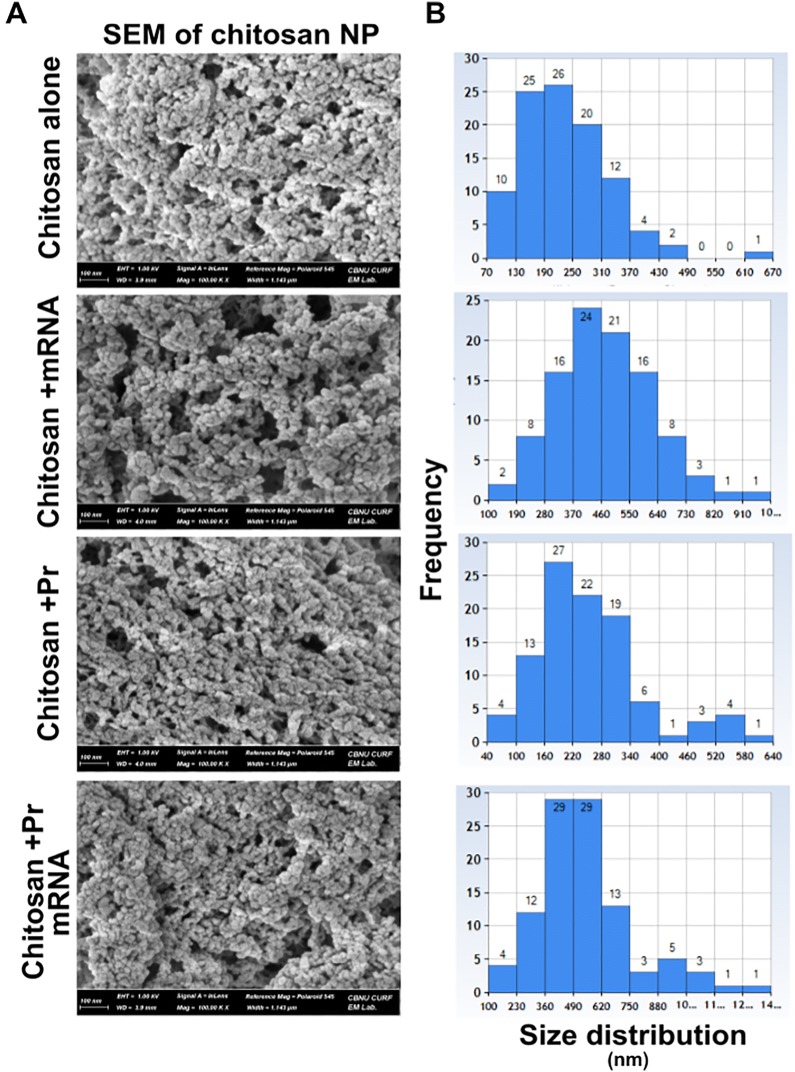
**Scanning Electron Microscopic characterization of CNPs decorated with HA2 and M2e recombinant proteins and mRNA. A** Field Emission Scanning Electron Microscope (FESEM) analysis of empty CNPs, RNA-CNPs, protein-CNPs, and RNA–protein CNPs. Each specimen was observed at 100 K magnification. **B** Particle size distribution was determined from randomly selected 100 particles on each image. Particle size was measured in “nm”.

The CNPs were prepared in a 4% acetic acid solution and the pH was adjusted to 4.5. At this pH, chitosan amine groups are highly protonated and can form highly stable electrostatic interactions with the negatively charged groups from HA2 and M2e mRNA molecules. To entrap mRNA molecules inside CNPs, negatively charged cross-linked sodium TPP was used. Since chitosan is a positively charged particle, electrostatic interactions helped to bind HA2-HA1 and M2e recombinant proteins to the surface of the CNPs (Figure 1). Our results indicated that the surface conjugation of HA2-HA1 and M2e proteins was around 50% (optical density ratio 1.2:1). Bound protein calculations revealed approximately 200 µg of CNPs entrapped 843 ng of HA2-HA1 protein and 641 ng of M2e respectively when individual proteins were interacted with CNPs (Additional file 1). These surface coated CNPs or the empty particles were characterized for their surface morphology by SEM and TEM analysis. Interaction of CNPs caused particle size variations that are clear by SEM imaging (at 100K × magnification) and particle size distribution analysis. The CNPs interacting either with RNA or proteins caused the formation of larger nanoparticles with an average particle size that varied between 100 and 800 nm. In contrast, naked CNPs were 70–490 nm in particle size after formulation and processing for analysis (Figure 2). The spherical appearance and the particle size were further confirmed by TEM analysis. The size of individual particle was less than 100 nm (Additional file 3).

### CNPs efficiently penetrate the nasal mucosal barrier and are rapidly taken up by chicken macrophages

**Figure 3:**
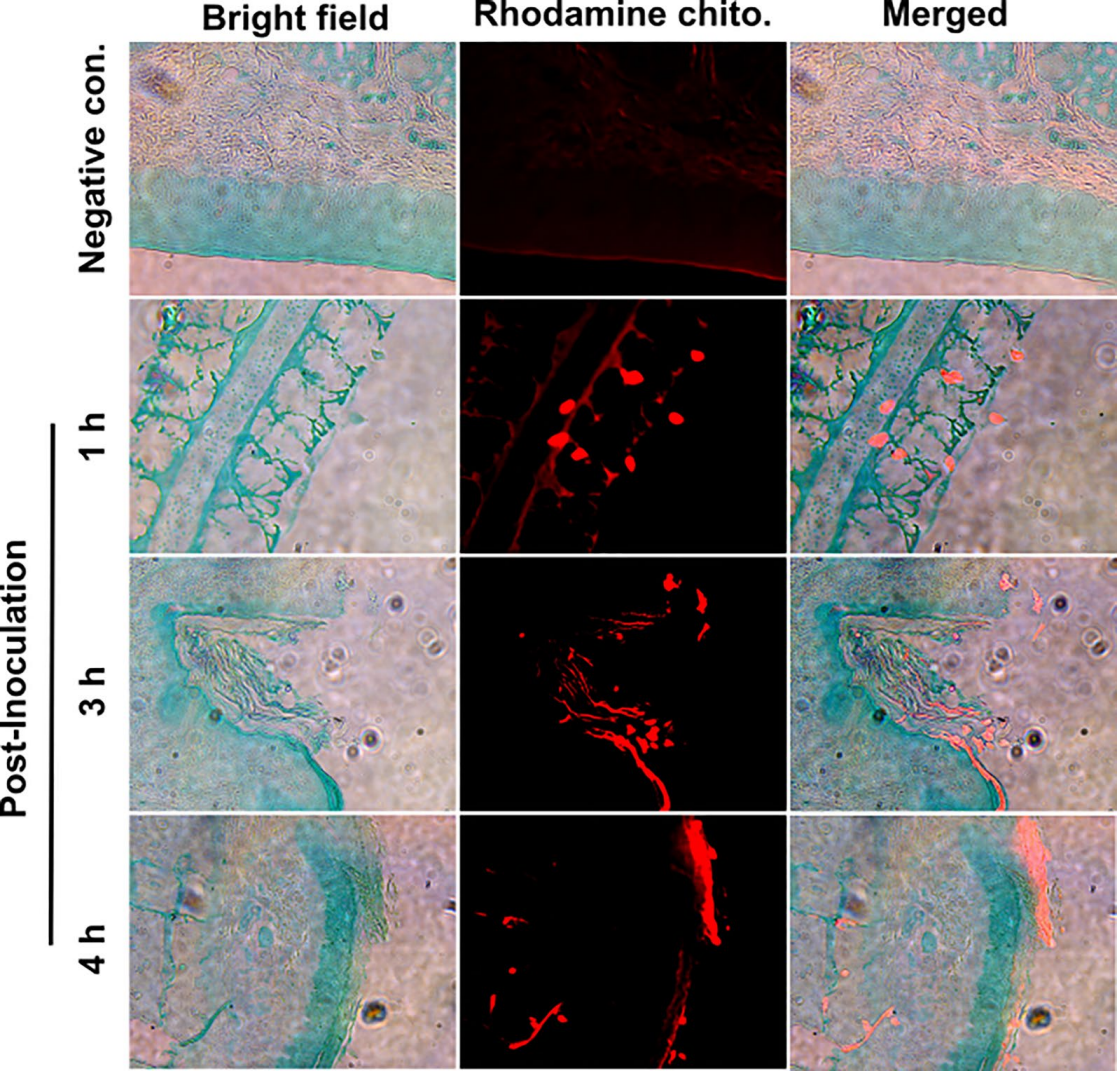
**In vivo analysis of CNPs retention in chicken nasopharyngeal tissues.** Female layer chickens (*n* = 3 in each group) were nasally treated with RITC tagged CNPs. Tissue sections were assessed 1 h, 3 h and 4 h after inoculation. Specific attachment of CNPs could be seen in red fluorescence. With time, CNPs penetrated deeper layers of the mucosal tissues. The stained tissues were visualized under a fluorescent microscope (Scale bar: 100 μm). Red fluorescent areas represent the penetration of CNPs inside intestinal tissues. The experiment was repeated twice.

To investigate the ability of CNPs to adhere to the mucosal surface of the nasopharyngeal region, chickens were inoculated via the nasal route with RITC tagged CNPs. One, three, and four hours after the initial administration, tissue sections from the nasal region were harvested and processed for IHC imaging under the fluorescence microscope. We observed the firm attachment of CNPs into the mucosal tissues even after 3 h. Further, chickens were observed 2 days after inoculation and the result revealed that CNPs were still present in the nasal mucosal tissue (data not shown). In the present study, to avoid artifacts that can be generated by CNP unbound RITC dye, CNPs were generated as a fine powder (grounded in the presence of liquid nitrogen and desiccated) and resuspended in PBS. Results could be observed as solid particles embedded in the mucosal tissues (Figure 3). We further confirmed the potential of chicken macrophages to take up CNPs. Our results indicated that fluorescently tagged empty CNPs were efficiently taken up by chicken macrophages, which showed less fluorescence (Additional file 4). These findings indicate that CNPs can be taken up by chicken immune cells and presented to activate adaptive immune responses.

### CNPs efficiently deliver mRNA molecules into APCs

**Figure 4:**
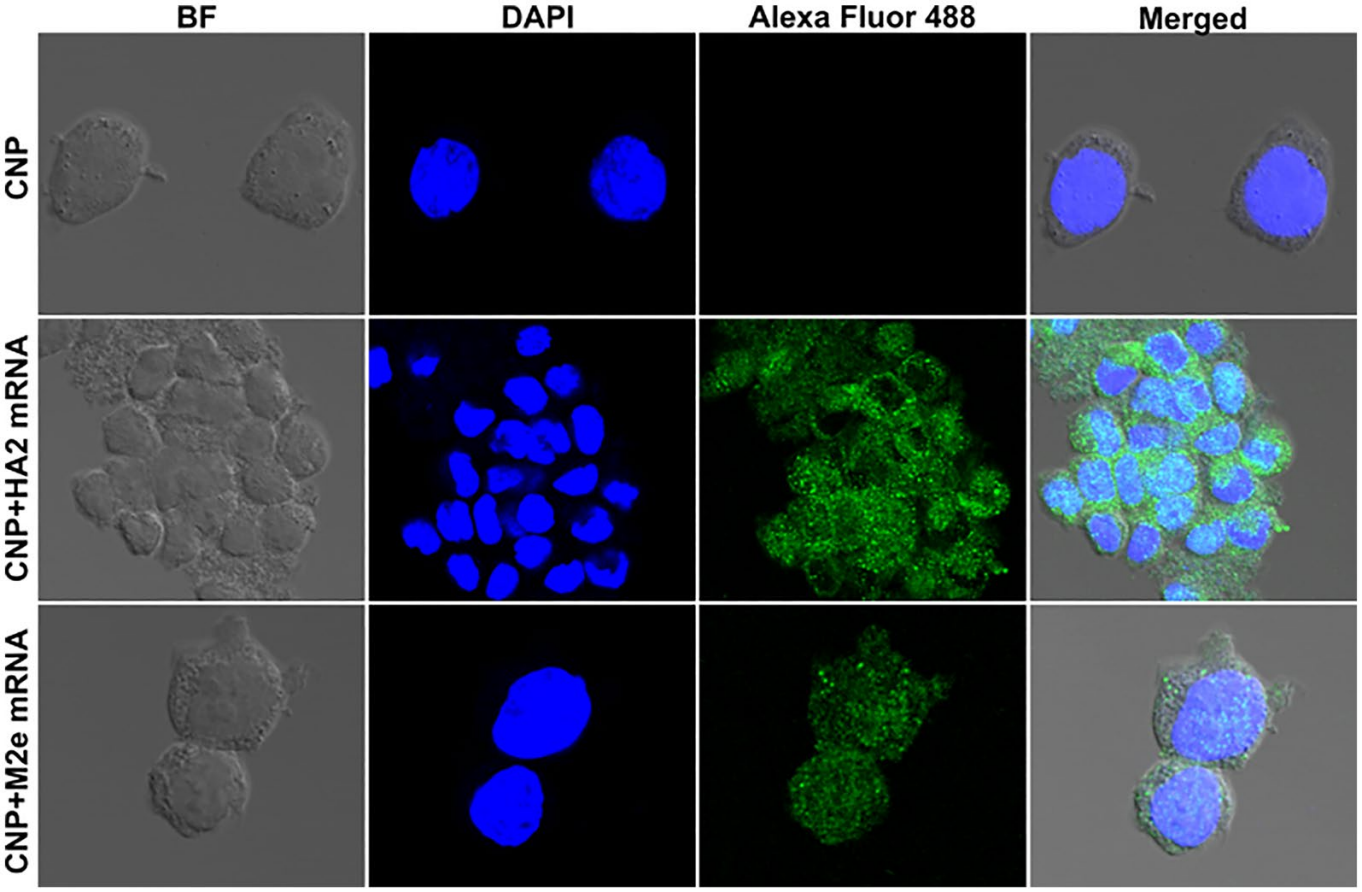
**HA2 and M2e mRNA cell delivery and expression by CNPs. THP-1 cells were treated with empty CNPs, CNPs delivering HA2 mRNA, or CNPs delivering M2e mRNA.** After 16 h, the cells were visualized under a confocal microscope (Scale bar: 20 µM). The experiment was repeated twice. The results shown represent one independent experiment.

We developed HA2 and M2e mRNA molecules encoding the respective proteins in a membrane-bound form. A human kappa immunoglobulin (IgK) signal peptide was incorporated into the mRNA vaccine transcripts for efficient translocation of proteins through the cell secretory network and cell surface expression [22]. To assess the capacity of CNPs to deliver entrapped mRNA molecules inside APCs, THP-1 cells were incubated with empty CNPs (approximately 250 µg CNPs), CNP-M2e mRNA, or CNP-HA2 mRNA. For comparison studies, we treated THP-1 cells with LyoVec, LyoVec-M2e mRNA or LyoVec-HA2 mRNA. Post-16 h incubation, cells were examined by fluorescent confocal microscopy. Our results indicated that CNPs efficiently deliver mRNA molecules into the mammalian cells as green fluorescence was evident in cells treated with either CNPs delivering M2e mRNA or HA2 mRNA molecules (Figure 4). Based on the intensity and the number of cells positive for the fluorescence, we observed that cells treated with CNPs delivering mRNA molecules exhibited greater intensity of fluorescence than the LyoVec mediated mRNA delivery (Additional file 5). Further, a greater proportion of cells showed green fluorescence in the case of CNPs delivering mRNA molecules, suggesting that CNPs represent an efficient antigen delivery system.

### CNPs produce no cytotoxic effects on chicken RBCs and lung cells

**Figure 5:**
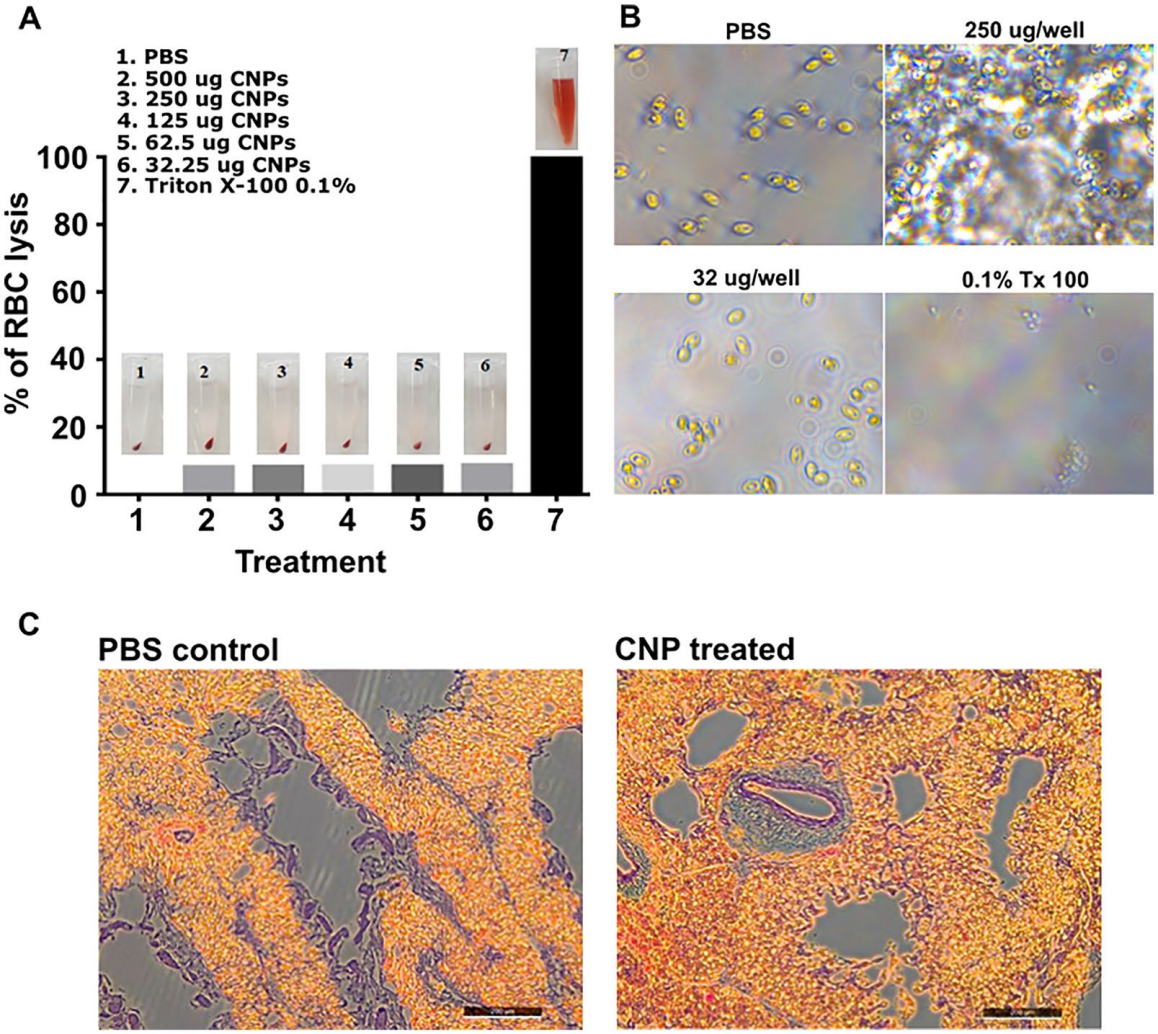
**In vitro and in vivo safety evaluation of CNPs. A** Hemolysis assay in chicken RBCs: 1. PBS control; 2–6, increasing concentrations of CNPs; 7, Triton-X 100 control. **B** Microscopy images of chicken RBCs incubated with PBS, different concentrations of CNPs, or Triton-X 100. **C** Effect of CNPs on chicken lungs. Chickens (*n* = 3 in each group) were intranasally administered with either PBS or CNPs, and after 5 days, their lungs were analyzed for histological changes. The experiment was repeated thrice. The results shown represent one independent experiment.

Hemolysis is a well-established method to assess the biocompatibility analysis for nanoparticles [38]. Biocompatibility was investigated by quantifying the effect of CNPs on the hemolysis of chicken RBCs. Our results indicated that there was no significant increase in the hemolysis percentage in the groups treated with CNPs compared to the group treated with PBS (Figure 5A). RBCs treated with Triton X-100 were found to be lysed, while RBCs treated with CNPs were intact under microscopical examination (Figure 5B). To further analyze the effect of CNPs in vivo, we intranasally administered CNPs in chickens and after 5 days post-administration, histological analysis was performed on treated lungs (Figure 5C). Our results demonstrated that CNPs treated lungs exhibited no abnormal histology and appeared comparable to those from the PBS treated group. These findings point out that CNPs are safe and can be administered intranasally without any adverse cytotoxicity issues.

### Incorporation of mRNA molecules enhances functional neutralizing antibody titers

**Figure 6:**
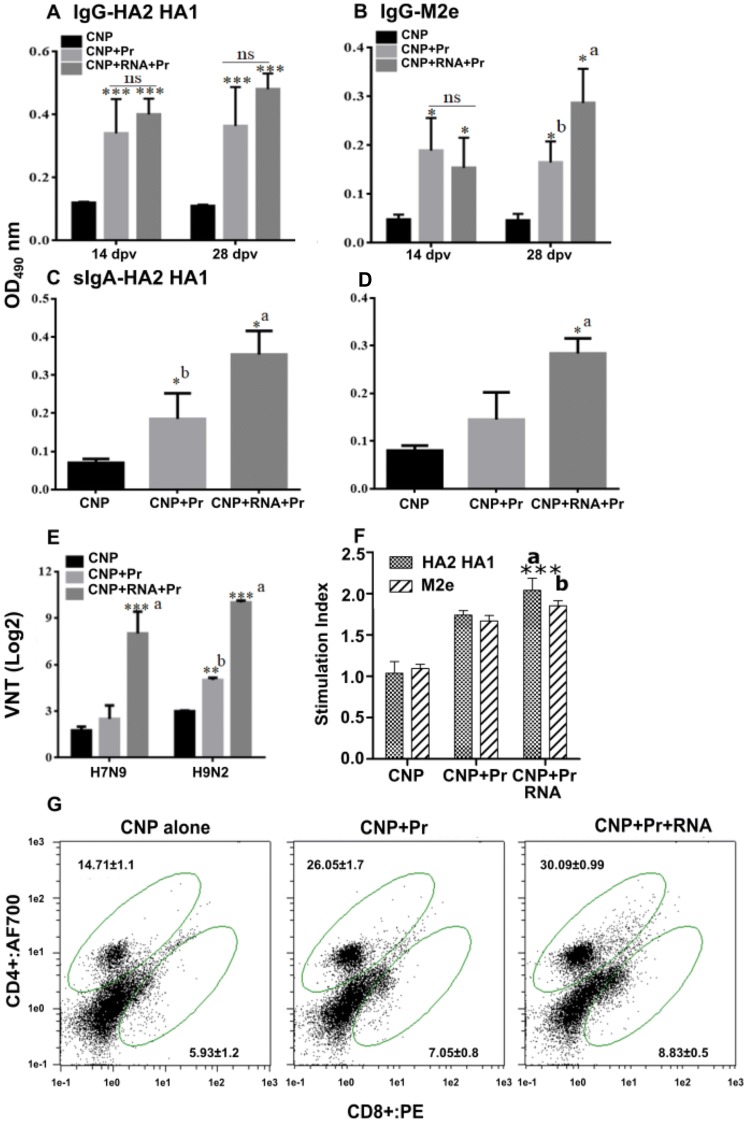
**Antigen-specific ELISA, VNT and cell mediated immune response.** Chickens (*n* = 17 in each group) were intranasally vaccinated with empty CNPs, CNPs delivering antigens in both protein (Pr) and mRNA formats (CNPs + mRNA + Pr) or CNPs delivering antigens in protein (Pr) format only (CNPs + Pr) and 3 weeks later chickens were boasted with the same dose and route. An indirect ELISA analyzed the serum IgG and mucosal IgA responses at 14 and 28 days post-first vaccination. **A** M2e-specific IgG responses. **B** HA2-HA1-specific IgG responses. **C** M2e-specific IgA responses. **D** HA2-HA1-specific IgA responses. **E** VNT post-challenge. Each data points represent mean ± SD of six chickens per group. *p < 0.05. **p < 0.01. ***p < 0.001. ns, non-significant. A significant concerning CNPs and CNPs + Pr; b, concerning CNPs only. **F** Lymphocyte proliferative responses against each antigen (*n* = 6), *** *p* < 0.001. **G** Flow cytometry analysis of CD4+ and CD8+ T-cell response against HA2-HA1 antigen. ***p < 0.001.

To investigate the effect of vaccination on systemic and mucosal antibody responses, indirect ELISA for IgG and IgA was performed post-vaccination in sera and lung wash samples, respectively. The M2e and HA2 specific IgG and IgA responses were induced following vaccination with CNPs delivering HA2 and M2e antigens (Figure 6). Chickens vaccinated with CNPs delivering HA2 and M2e antigens in both protein and mRNA formats elicited higher systemic and mucosal antibody responses compared to the chickens that received CNPs delivering antigens only in protein format, yet the difference was not always statistically significant. While M2e-specific IgG responses were almost comparable at the measured time-points (Figure 6A), HA2-specific IgG responses at 28^th^-day post-vaccination were significantly (*p* < 0.05) higher in chickens vaccinated with CNPs delivering antigens in both protein and mRNA formats (Figure 6B). We next analyzed IgA responses in lung washings at day 28 post-first vaccination. Our results demonstrated that chickens vaccinated with CNPs delivering HA2 and M2e antigens in both protein and mRNA formats elicited significantly (*p* < 0.05) higher HA2 and M2e-specific IgA responses than CNPs delivering antigens in protein format only (Figures 6C and D).

We next analyzed virus neutralization titers (VNT) among vaccinated chickens post H7N9 and H9N2 challenge by serum microneutralization assay (Figure 6E). Our results showed that VNT were higher in vaccinated chickens compared to the control group, yet the difference was not always statistically significant (Figure 6). Concerning the H7N9 challenge, our results indicated that chickens that received CNPs delivering antigens in both protein plus mRNA formats elicited significantly (*p* < 0.001) higher VNT compared to the group that received CNPs delivering protein only, which showed VNT comparable to that of the control group challenged with the H7N9 virus. Concerning the H9N2 challenge, both vaccinated groups showed significantly (*p* < 0.05) higher VNT than H9N2 challenged control group. Among vaccinated groups, chickens that received CNPs delivering antigens in both protein and mRNA formats elicited significantly (*p* < 0.001) higher titers than CNPs delivering protein only. These results indicate that the incorporation of mRNA molecules significantly enhanced functional protective antibody responses.

### Cell-mediated immune response

The cellular immune responses were evaluated by determining the lymphocyte proliferation response (Figure 6F) and changes in CD4+ and CD8+ T-cell populations upon re-stimulation with the CNP delivered antigens (Figure 6G). The CMI responses were assessed against both HA2-HA1, and M2e antigens and revealed significantly higher response towards HA2-HA1 antigen than M2e antigen. This observation can be an outcome of higher coating efficacy of HA2-HA1 antigen than the M2e. The higher surface for interaction in HA2-HA1 protein may increase the interaction with CNPs than the smaller protein M2e causing enhanced adsorption and leading to enhanced HA2-HA1 specific response. Flow cytometry analysis revealed that immunization with CNPs delivering proteins and mRNAs particularly induced CD4+ T-cell population along with a moderated increase in CD8+ T-cell response. However, both types of response can be highly encouraged for mucosal immunity that involves both humoral and cell-mediated immunity.

### Incorporation of mRNA molecules enhances the protective efficacy of protein-coated CNP-based vaccine

**Figure 7:**
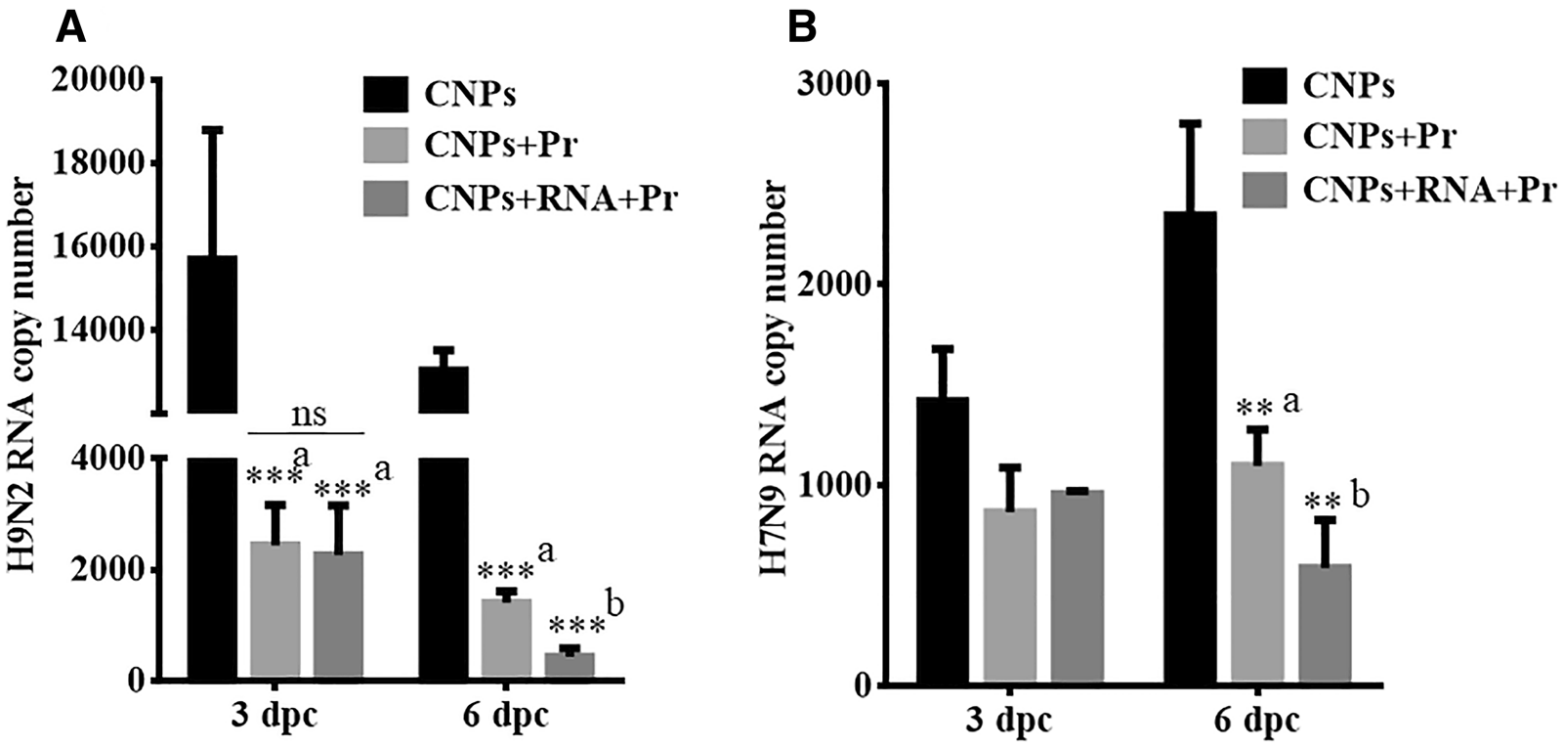
**Protective efficacy of the CNP-based HA2-HA1 + M2e vaccine against LPAI viruses.** Chickens (*n* = 17 in each group) were intranasally vaccinated with empty CNPs, CNPs delivering antigens in both protein (Pr) and mRNA formats (CNPs + mRNA + Pr) or CNPs delivering antigens in protein (Pr) format only (CNPs + Pr) and 3 weeks later chickens were boasted with the same dose and route. At 28 days post-first vaccination, all the vaccinated chickens were challenged with 10^4^ TCID_50_ of either H7N9 or H9N2 virus. The protective efficacy was determined by the estimation of viral RNA copy numbers in the cloacal swab samples of the immunized chickens (*n* = 5) after the challenge with the virulent AIV. **A** Protection efficacy against the H9N2 challenge. **B** Protection efficacy against the H9N2 challenge. **p < 0.01. ***p < 0.001. ns, non-significant. a, with respect to CNPs only; b, significant with respect to CNPs and CNPs + Pr.

To determine the protective efficacy of a CNP-HA2-M2e based influenza vaccine, we intranasally challenged all the vaccinated and the control chickens with a virulent dose (10^4^ TCID_50_) of either H7N9 or H9N2 virus. Subsequently, cloacal swab samples were collected post-challenge for the determination of viral RNA copy number by qRT-PCR assay (Figure 7). The presence of viral RNA was found in all the groups from day 1 to 6 post-challenge, albeit, vaccinated chickens exhibited significantly (*p* < 0.01) lower viral load than the control chickens. Among vaccinated chickens, we showed that chickens vaccinated with CNPs + RNA + Pr showed significantly (*p* < 0.001) lower viral load at day 6 post-H9N2 challenge compared to the chickens that were vaccinated with CNPs + Pr. The H9N2 viral load found on day 3 post-challenge was comparable in both the vaccination groups (Figure 7A). We next investigated the potential of conserved epitopes to stimulate heterologous protection against the H7N9 challenge. Although both the vaccination groups showed a lower viral load than control chickens, a statistically significant difference was only observed on the day 6 post-H7N9 challenge (Figure 7B). Furthermore, on day 6 post-challenge comparable viral loads were found in both vaccination groups.

**Figure 8:**
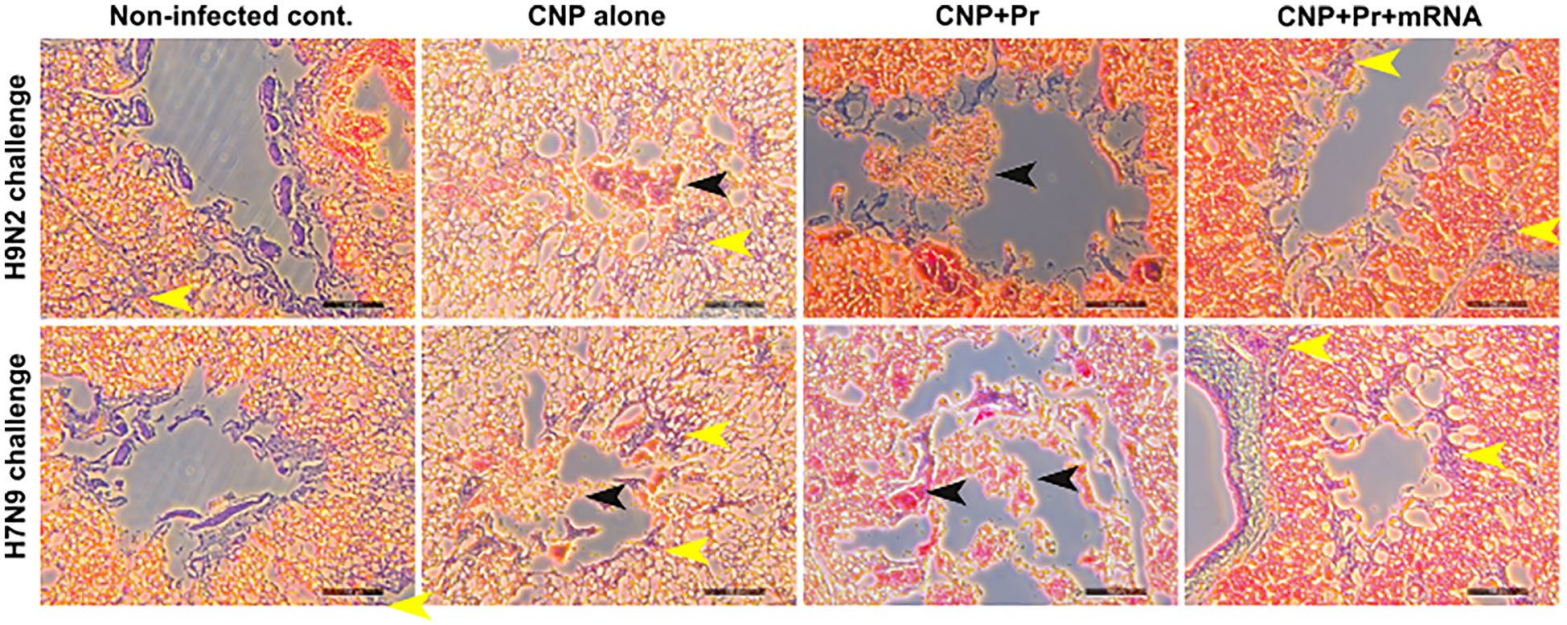
**Photomicrographs of hematoxylin-and eosin-stained lung sections of chickens on 8**^**th**^**day post-challenge.** Chickens (*n* = 17 in each group) were intranasally vaccinated with empty CNPs, CNPs delivering antigens in both protein (Pr) and mRNA formats (CNPs + mRNA + Pr) or CNPs delivering antigens in protein (Pr) format only (CNPs + Pr) and 3 weeks later chickens were boasted with the same dose and route. At 28 days post-first vaccination, all the vaccinated chickens were challenged with 10^4^ TCID_50_ of either H7N9 or H9N2 virus. Chickens that received empty CNPs showed hyperemia with infiltration of mononuclear inflammatory cells in bronchi and lung parenchyma, while birds vaccinated with CNPs delivering antigens in protein format only showed significantly lower inflammatory lesions than CNPs alone. Lungs of chickens vaccinated with CNPs delivering antigens in both formats appeared like that of the uninfected lungs. Scale bar = 100 µM. Each picture is representative of 6 chickens. Black arrows indicate fluid filed areas of the lung tissues and yellow arrows represent macrophages infiltrated in the lung tissues due to infection.

To further investigate the effect of vaccination on virus-specific immune protection, histopathological studies were performed on lung tissues collected from birds on day 8 post-H7N9 or H9N2 challenge (Figure 8). As expected, no lesion was found in the lung tissues of uninfected chicken. Chickens treated with CNPs alone had signs of inflammation, macrophage infiltration, congestion, and hemorrhagic exudates in the lungs. Compared to control, chickens vaccinated with CNPs + Pr exhibited lower inflammatory lesions post H7N9 or H9N2 challenges. However, chickens that received CNPs + RNA + Pr considerably inhibited virus-induced lung pathology and the lungs histology appeared normal to that of the uninfected chickens. These findings clearly support the conclusion that mRNA had significantly augmented the immunogenicity of the subunit-based vaccines.

## Discussion

The induction of mucosal immune responses in the respiratory tract represents an effective vaccination strategy for protection against influenza virus infection. Thus, mucosal adjuvants, such as cholera toxin and *E. coli* heat-labile toxin, have been previously employed to elicit effective immune protection against influenza [39, 40]. However, these potent mucosal adjuvants have been associated with toxicity issues, including severe diarrhea and central nervous system disorders [41, 42]. Therefore, the development of safer and effective mucosal adjuvants is necessary for the delivery of vaccine antigens via the mucosal route. Chitosan, a cationic polysaccharide derived from chitin, is considered as a safe and biocompatible polymer [28]. In the present study, we prepared CNPs entrapping HA2-HA1 mRNA and surface interacting each protein. Hereby we anticipate a rapid presentation of antigens loaded on the surface of CNPs and subsequent release of mRNA into the APC upon particle acquisition. This procedure may mimic the natural order of influenza virions. The present study demonstrated that CNPs had no significant effect on the viability of chicken RBCs and intranasally administered CNPs resulted in no distinct alteration in lung histology compared to the chickens that received sterile PBS. These findings are in agreement with the previously published reports, which show that chitosan is safe and can be administered nasally in mouse model [28]. Nanoparticle characteristics including size, shape and surface charge, determine their biological properties [43]. The prepared CNPs had almost a uniform size dispersion, were spherical and less than 100 nm in size. These tiny particles interact with each other and form large particles when interacting with RNA or proteins. Our in vitro studies demonstrated that these particles were readily taken by antigen presenting cells such as macrophages and this finding was in agreement with the previously published report, which shows that particles less than 200 nm are readily uptaken by APCs via pinocytosis [44]. Furthermore, our study shows that CNPs efficiently deliver mRNA molecules into monocytes compared to the LyoVec mediated mRNA delivery. Thus, CNPs represent an efficient delivery system capable of targeting immunogenic antigens to the APCs, which subsequently stimulate a more protective adaptive immune system.

In the present study, encapsulation of mRNA molecules of HA2 and M2e within CNPs was followed by surface coating with conserved influenza proteins. The protein binding efficiency is dependent on the charge of the cargo proteins, which is related to the isoelectric point (pI), a pH at which protein carries zero net charge. Thus, increasing the pH above the pI makes the protein being negatively charged. We resuspended HA2-HA1 (pI 5.75) and M2e (pI 3.66) proteins in PBS (pH = 7.4). These proteins were negatively charged, thus facilitating highly stable interactions with the cationic chitosan polymer. We found that the formulated CNPs had a high protein loading efficiency which was consistent with earlier reports [45, 46]. Our protein adsorption study revealed HA2-HA1 has a higher coating efficacy than M2e, possibly due to a larger molecule with the higher surface for interaction with CNPs. Further, when CNPs were incubated with a combination of proteins together at once, competition between two proteins can be found and the M2e was coated in lesser quantities than the HA2-HA1. Specific properties and ionic charge of each molecule may be affecting this observation. Previous studies show that chitosan has self-assembling abilities and can adhere to the mucosal surfaces for the delivery of immunogenic antigens to the immune cells [28, 47], which is a prerequisite for efficient induction of antigen-specific humoral and cell-mediated immunity. The present study demonstrated that CNPs efficiently bound to the epithelial cells of intestines and lungs and effectively crossed the epithelial barrier to penetrate deeper into the tissues. This suggests that CNPs are likely to be taken by the APCs of the mucosal immune system, which subsequently carries delivered antigens to the immune initiation sites, such as Peyer’s patches, for efficient induction of immune responses.

Intranasal administration of vaccines is the logical route to control infections caused by IAVs and has many advantages, especially for mass implementation of vaccination, thereby preventing the occurrences of cross-contamination of blood-borne pathogens because of the parenteral injection and needle re-use. Furthermore, intranasal vaccination is needle-free, which seems to be a logistic approach in pandemic situations, when time and trained medical personnel could be the limiting factors. To overcome the problems of antigenic diversity among influenza subtypes, considerable studies have investigated the potential of conserved HA stalk domain and M2e antigens to elicit a broad spectrum of protection against various influenza subtypes [17, 48]. The sequence analysis of influenza virus subtypes revealed that the HA2 part is considerably more conserved than the HA1 part. The present immunogen HA2 is based on the sequence of H9N2, and phylogenetic analysis of HA2 sequences from H7N9 and H9N2 LPAI viruses revealed around 60% sequence similarity. Earlier studies have shown that monoclonal antibody C179 directed against the conserved epitope in the HA stem region can neutralize influenza viruses belonging to various subtypes [49, 50]. Furthermore, previous studies have shown that monoclonal antibodies directed to the closely related epitopes on the HA stem region can mediate neutralization of divergent influenza viruses, suggesting that the HA2 antigen represents an attractive choice to evoke broadly protective neutralizing Abs [51–53]. Besides HA2, several major studies have demonstrated the potential of M2e-based vaccines to provide heterologous protection against various subtypes of influenza A viruses [48]. A study by Song et al. showed that prophylactic and therapeutic administration of M2e-specific Z3G1 monoclonal antibody resulted in significant protection in mice and alleviated clinical symptoms and lung pathology in monkeys following H1N1 infection [54]. Following this notion, we coadministered HA2 and M2e antigens to induce a broad spectrum of protection against LPAI viruses. Recently, mRNA-based vaccines have shown promising results and have demonstrated their potential to provide complete protection against lethal infections. Thus, the immunization strategy of administering immunogenic antigens in both mRNA and protein formats might represent an effective and promising approach to elicit superior immune responses against infectious diseases. Previous studies have reported that *E. coli* expressed influenza proteins require efficient adjuvants to stimulate protective mucosal immune responses, particularly IgA production [55, 56]. Chitosan represents an efficient antigen delivery system and previous studies have demonstrated its potential to augment the immunogenicity of vaccine antigens that are delivered via mucosal route [28, 57]. Several studies have explored the intranasal administration of M2e- and HA2-based vaccines and compared the effectiveness of this route with other routes of vaccination [21, 58]. Hervé et al. showed that intranasal administration of recombinant nucleoprotein of respiratory syncytial virus substituted with three tandem copies of M2e elicited both M2e-specific systemic and mucosal antibody responses and efficient protection against PR8 challenge compared to the subcutaneous administration, which induced only IgG responses [59]. Another study has shown that intranasal immunization with M2e plus HA2 conjugated with cholera toxin subunit B induced efficient and broad spectrum of immunity against influenza viruses compared to parenteral administration [58]. The present study demonstrated that intranasally administered CNPs loaded with HA2 and M2e antigens in both mRNA and protein formats elicited efficient systemic and mucosal antibody responses and provided homo- and heterologous protection against LAPI viruses. Induction of HA2 and M2e-specific IgG responses in peripheral blood circulation is the principal mode of protection mediated by HA2 and M2e-based vaccines [21, 58], while the protection mediated by anti-M2e IgA antibodies remains largely unclear. A study by Renegar et al. reported that both IgG and IgA antibodies are important, with plasma IgG serving as the back-up for secretory IgA-mediated protection in the nasal compartment, and IgG being the dominant antibody in the protection of the lung [60]. We found that incorporation of mRNAs in the vaccine formulation enhanced protective immune responses elicited by HA2-HA1 and M2e recombinant proteins. Our data showed that both IgG and IgA responses were superior in chickens vaccinated with CNPs delivering antigens in both protein and mRNA formats. This might explain the efficient protection observed against LPAI viruses offered by protein-coated CNPs entrapping mRNA molecules. We further demonstrated that the incorporation of mRNAs in the vaccine formulation elicited superior neutralizing antibodies against H7N9 and H9N2 viruses. In addition to antibody-mediated humoral responses, nasal immunization with CNPs were eliciting CMI responses marked by lymphocyte proliferation responses. Further, proficient CD4+ T-cell response and moderate CD8+ T-cell response also demarcate the specific engagement of Th2 and Th1 type immune responses that play a vital role against viral infections. Consequently, chickens vaccinated with CNPs delivering antigens in both protein and mRNA formats considerably reduced viral shedding in feces and exhibited very low lung pathology compared to chickens treated with CNPs delivering antigens in protein formats. Although it remained unclear, at least in this study, the individual role played by each influenza conserved protein in conferring protection against each subtype of AIV; our aim was to deliver the HA2 and M2e antigens as a co-mix, which has been previously reported a superior vaccination strategy [61].

In conclusion, we show that intranasally delivered CNPs loaded with influenza HA2 and M2e antigens elicited efficient protective immune responses against LPAI H7N9 and H9N2 viruses. We show that the incorporation of mRNAs in the vaccine formulation provides an efficient tool to generate more potent neutralizing antibodies against infectious diseases. In addition to dose optimization, further studies are warranted to investigate the potential role played by each HA2 and M2e antigen in eliciting protection against AIVs.

## Supplementary information

**Additional file 1. Adsorption of HA2-HA1 and M2e into CNPs.** Interaction of each individual HA2-HA1 and M2e proteins with CNPs were determined by ELISA using suspended CNPs in coating buffer. Specific presence of each antigen was determined by using antigen specific polyclonal antibodies. After conducting ELISA, the variation of signal intensity was compared and the ratio between two proteins were considered as the estimation of relative proportion present on CNPs.

**Additional file 2. SDS-PAGE and Western blot analysis of HA2-HA1 protein.** (A) The SDS-PAGE analysis of HA2-HA1 protein. (B) Western blot analysis using polyclonal HA-specific antibody. Lane M, protein marker (Cat No. #P8501-020) and lane 1, HA2-HA1 protein.

**Additional file 3. TEM analysis of protein-coated CNPs.** CNPs were prepared by an ion gelation method followed by surface coating with HA2-HA1 plus M2e proteins. The surface-coated CNPs were visualized under an electron microscope. Arrows represent surface bounded recombinant proteins.

**Additional file 4. Chicken macrophages efficiently take up CNPs in vitro.** Macrophages were treated with RITC aged CNPs. RITC tagged CNPs were made into a fine powder and resuspended in PBS. The specific presence of fine particles within macrophages was visualized under a fluorescent microscope (Scale bar: 100 μm). The experiment was repeated twice and the results shown are representative images.

**Additional file 5. Confirmation of mRNA expressions of HA2-HA1 and M2e in mammalian cells.** THP-1 cells were treated with LyoVec delivering HA2 mRNA (A), LyoVec delivering M2e mRNA (B), or LyoVec alone (C), and after 16 h cells were visualized under a confocal microscope (Scale bar: 20 µM). The experiment was repeated twice and the results are one independent experiment.

## Data Availability

The data used and analyzed during the current study can be made available upon reasonable request.

## Abbreviations

CNP: chitosan nanoparticles
Pr: protein
AIV: avian influenza virus
HPAI: highly pathogenic avian influenza
LPAI: low pathogenic avian influenza
HA: hemagglutinin
M2: matrix protein 2
M2e: matrix protein 2 ectodomain
