# Supplementary material for: Intranasally administered protein coated chitosan nanoparticles encapsulating influenza H9N2 HA2 and M2e mRNA molecules elicit protective immunity against avian influenza viruses in chickens
Source: Vet Res. 2020 Mar 6;51:37. doi: 10.1186/s13567-020-00762-4 (PMC7060564; doi:10.1186/s13567-020-00762-4)
Supplement: Supplementary file 1 — Additional file 1. Adsorption of HA2-HA1 and M2e into CNPs. Interaction of each individual HA2-HA1 and M2e proteins with CNPs were determined by ELISA using suspended CNPs in coating buffer. Specific presence of each antigen was determined by using antigen specific polyclonal antibodies. After conducting ELISA, the variation of signal intensity was compared and the ratio between two proteins were considered as the estimation of relative proportion present on CNPs. [file 13567_2020_762_MOESM1_ESM.docx]

**Additional file 1 Determination of HA2-HA1 and M2e chitosan bound protein ratio.** Protein coated chitosan nanoparticles were prepared as described in the manuscript. Chitosan nanoparticles were then re-suspended in 1 mL PBS. Then twenty microliters of the suspension per well was measured into a 96 well ELISA plate and 100 microliters of coating buffer was added and coated at 4 °C overnight. In the following day, plates were blocked with 5% BSA (Bovine Serum Albumin) and blocked for 1 h at room temperature. After washing three times with 1x PBS, the wells were reacted with antigen specific polyclonal antibodies at 1:3000 dilution ratio. After two hours of incubation, wells were washed with PBS for three times and color development was achieved by reacting with HRP tagged antibodies. Finally, the substrate O-Phenylenediamine dihydrochloride was added (OPD) and the color development was quantified at 490 nm wavelength using an ELISA plate reader. Average of three trials were used for calculation.

| **Absorbance at 490 nm** | | |  |  |  |  |  |
| --- | --- | --- | --- | --- | --- | --- | --- |
| **Antigen** | **100 ng** | **200 ng** | **300 ng** | **400 ng** | **500 ng** | **800 ng** | **1000 ng** |
| **HA2 HA1** | 0.1452 | 0.2444 | 0.49661 | 0.5637 | 0.620845 | 0.670506 | 0.60473 |
|  | 0.1431 | 0.2463 | 0.5543 | 0.63623 | 0.637437 | 0.6346 | 0.688467 |
|  | 0.1427 | 0.245 | 0.51579 | 0.5515 | 0.63845 | 0.78554 | 0.69479 |
| **Ave** | 0.143667 | 0.245233 | 0.522233 | 0.58381 | 0.632244 | 0.696882 | 0.662662 |
| **Stdev** | 0.001343 | 0.000971 | 0.02938 | 0.045805 | 0.009885 | 0.078851 | 0.05027 |

| **Absorbance at 490 nm** | | |  |  |  |  |  |
| --- | --- | --- | --- | --- | --- | --- | --- |
| **Antigen** | **100 ng** | **200 ng** | **300 ng** | **400 ng** | **500 ng** | **800 ng** | **1000 ng** |
| **M2e** | 0.09427 | 0.20443 | 0.315437 | 0.56295 | 0.560946 | 0.53623 | 0.537437 |
|  | 0.143 | 0.3431 | 0.408645 | 0.57587 | 0.571044 | 0.515 | 0.53845 |
|  | 0.10438 | 0.3242 | 0.389955 | 0.578554 | 0.569479 | 0.57547 | 0.529459 |
| **Ave** | 0.113883 | 0.290577 | 0.371346 | 0.572458 | 0.567156 | 0.542233 | 0.535115 |
| **Stdev** | 0.025717 | 0.075201 | 0.049312 | 0.008343 | 0.005435 | 0.030679 | 0.004925 |

| **Ratio calculation** | |  |  |  |  |  |  |
| --- | --- | --- | --- | --- | --- | --- | --- |
| **Antigen** | **100 ng** | **200 ng** | **300 ng** | **400 ng** | **500 ng** | **800 ng** | **1000 ng** |
| **HA2 HA1** | 0.143667 | 0.245233 | 0.522233 | 0.58381 | 0.632244 | 0.696882 | 0.662662 |
| **M2e** | 0.113883 | 0.290577 | 0.371346 | 0.572458 | 0.567156 | 0.542233 | 0.535115 |
| **Ratio** | 1.261525 | 0.843954 | 1.406327 | 1.01983 | 1.114761 | 1.285207 | 1.238354 |

Approximate ratio of HA2-HA1: M2e was 1.2:1.0 according to the present estimation.
