# Supplementary figures and images for: Intranasally administered protein coated chitosan nanoparticles encapsulating influenza H9N2 HA2 and M2e mRNA molecules elicit protective immunity against avian influenza viruses in chickens
Source: Vet Res. 2020 Mar 6;51:37. doi: 10.1186/s13567-020-00762-4 (PMC7060564; doi:10.1186/s13567-020-00762-4)

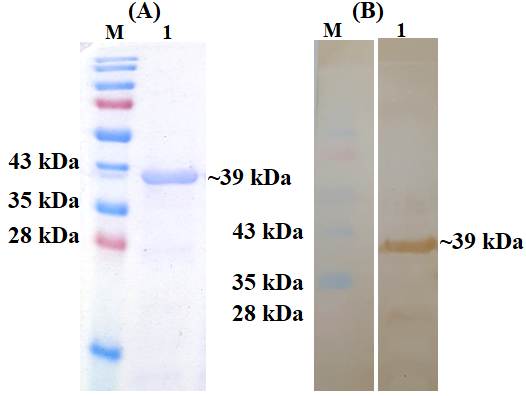

Supplement: Supplementary file 2 — Additional file 2. SDS-PAGE and Western blot analysis of HA2-HA1 protein. (A) The SDS-PAGE analysis of HA2-HA1 protein. (B) Western blot analysis using polyclonal HA-specific antibody. Lane M, protein marker (Cat No. #P8501-020) and lane 1, HA2-HA1 protein. [file 13567_2020_762_MOESM2_ESM.tif]

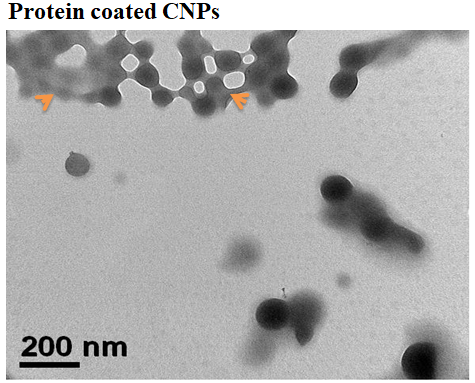

Supplement: Supplementary file 3 — Additional file 3. TEM analysis of protein-coated CNPs. CNPs were prepared by an ion gelation method followed by surface coating with HA2-HA1 plus M2e proteins. The surface-coated CNPs were visualized under an electron microscope. Arrows represent surface bounded recombinant proteins. [file 13567_2020_762_MOESM3_ESM.tif]

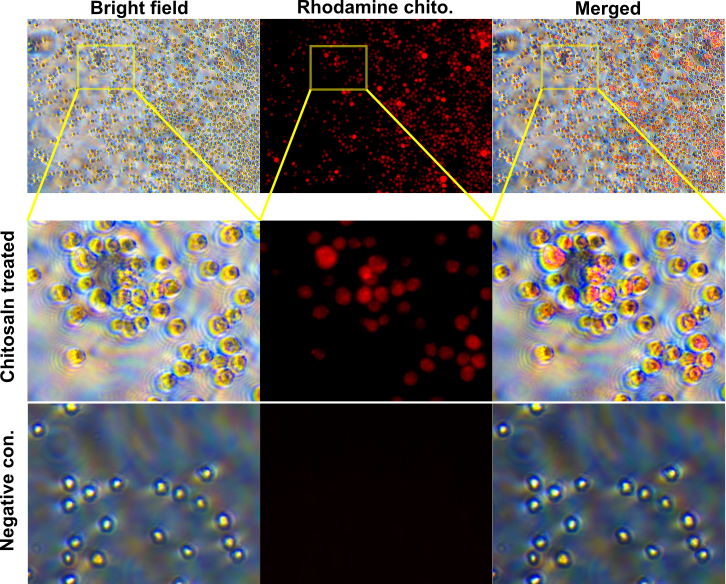

Supplement: Supplementary file 4 — Additional file 4. Chicken macrophages efficiently take up CNPs in vitro. Macrophages were treated with RITC aged CNPs. RITC tagged CNPs were made into a fine powder and resuspended in PBS. The specific presence of fine particles within macrophages was visualized under a fluorescent microscope (Scale bar: 100 μm). The experiment was repeated twice and the results shown are representative images. [file 13567_2020_762_MOESM4_ESM.tif]

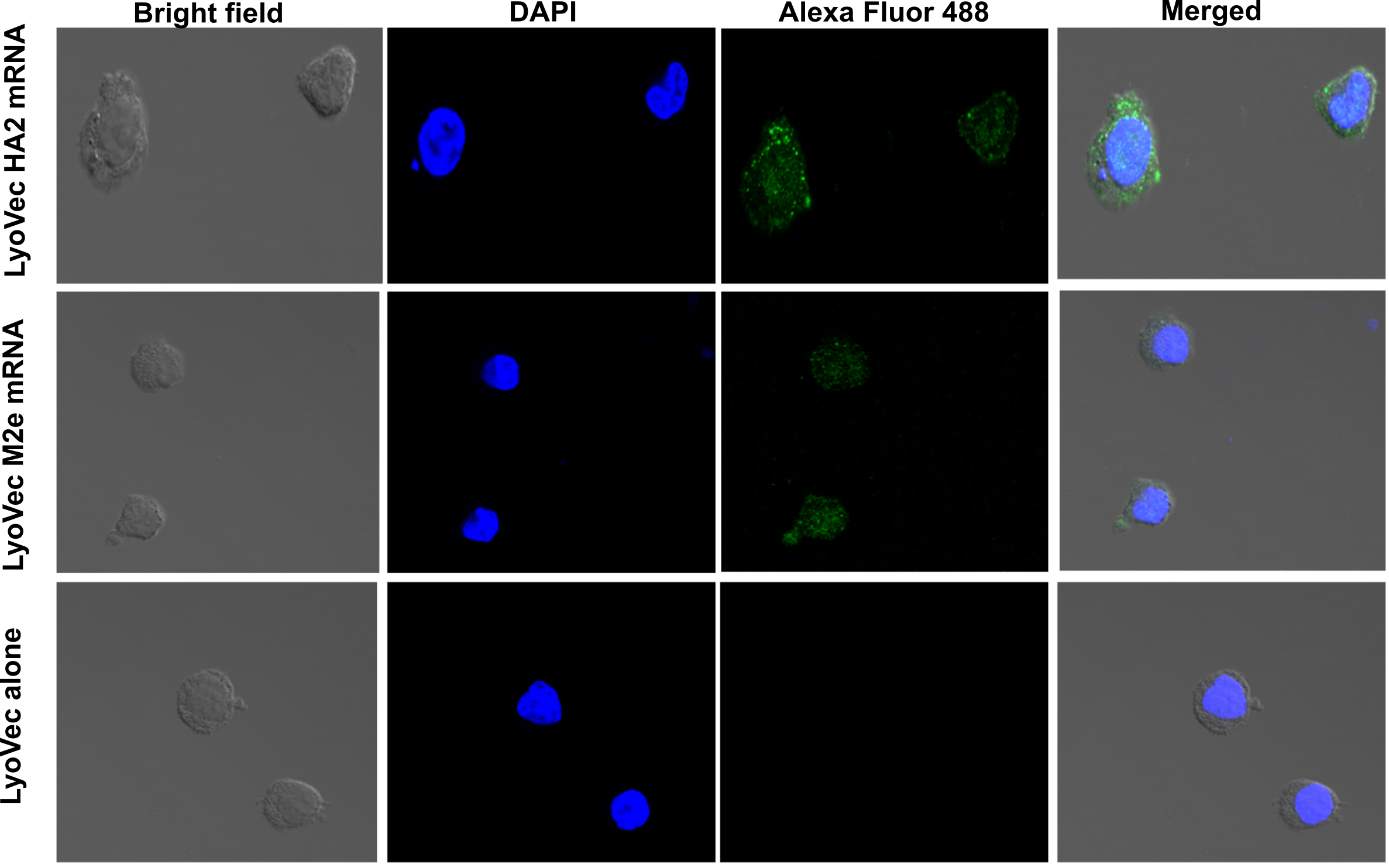

Supplement: Supplementary file 5 — Additional file 5. Confirmation of mRNA expressions of HA2-HA1 and M2e in mammalian cells. THP-1 cells were treated with LyoVec delivering HA2 mRNA (A), LyoVec delivering M2e mRNA (B), or LyoVec alone (C), and after 16 h cells were visualized under a confocal microscope (Scale bar: 20 µM). The experiment was repeated twice and the results are one independent experiment. [file 13567_2020_762_MOESM5_ESM.tif]
